# Human pluripotent-stem-cell-derived organoids for drug discovery and evaluation

**DOI:** 10.1016/j.stem.2023.04.011

**Published:** 2023-05-04

**Authors:** J. Jeya Vandana, Cassandra Manrique, Lauretta A. Lacko, Shuibing Chen

**Affiliations:** 1 Department of Surgery, Weill Cornell Medicine, 1300 York Avenue, New York, NY 10065, USA; 2 Center for Genomic Health, Weill Cornell Medicine, 1300 York Avenue, New York, NY 10065, USA; 3 Tri-Institutional PhD Program in Chemical Biology, Weill Cornell Medicine, The Rockefeller University, Memorial Sloan Kettering Cancer, New York, NY, USA; 4 Weill Cornell Graduate School of Medical Sciences, Weill Cornell Medical College, New York, NY, USA

## Abstract

Human pluripotent stem cells (hPSCs) and three-dimensional organoids have ushered in a new era for disease modeling and drug discovery. Over the past decade, significant progress has been in deriving functional organoids from hPSCs, which have been applied to recapitulate disease phenotypes. In addition, these advancements have extended the application of hPSCs and organoids for drug screening and clinical-trial safety evaluations. This review provides an overview of the achievements and challenges in using hPSC-derived organoids to conduct relevant high-throughput, high-contentscreens and drug evaluation. These studies have greatly enhanced our knowledge and toolbox for precision medicine.

## Teaser

Vandana and colleagues present a comprehensive review of the recent progress in using human pluripotent stem cells (hPSCs) and organoids for drug screening and clinical-trial safety evaluations. They also discuss the challenges and advancement associated with using hPSC-derived organoids for relevant high-throughput, high-content screens and drug evaluations.

## Introduction

Human pluripotent stem cells (hPSCs), which include human embryonic stem cells (hESCs) and induced pluripotent stem cells (iPSCs), are defined by their ability to both self-renew and differentiate into almost all terminally differentiated cells in the human body. hESCs were first derived in 1998 from the inner cell mass of pre-implantation embryos.^1^ In 2006, mouse fibroblasts were reprogrammed into pluripotent cells known as iPSCs.^2^ Similarly, human iPSCs (hiPSCs) were derived shortly after.^2^^,^^3^

**Figure 1:**
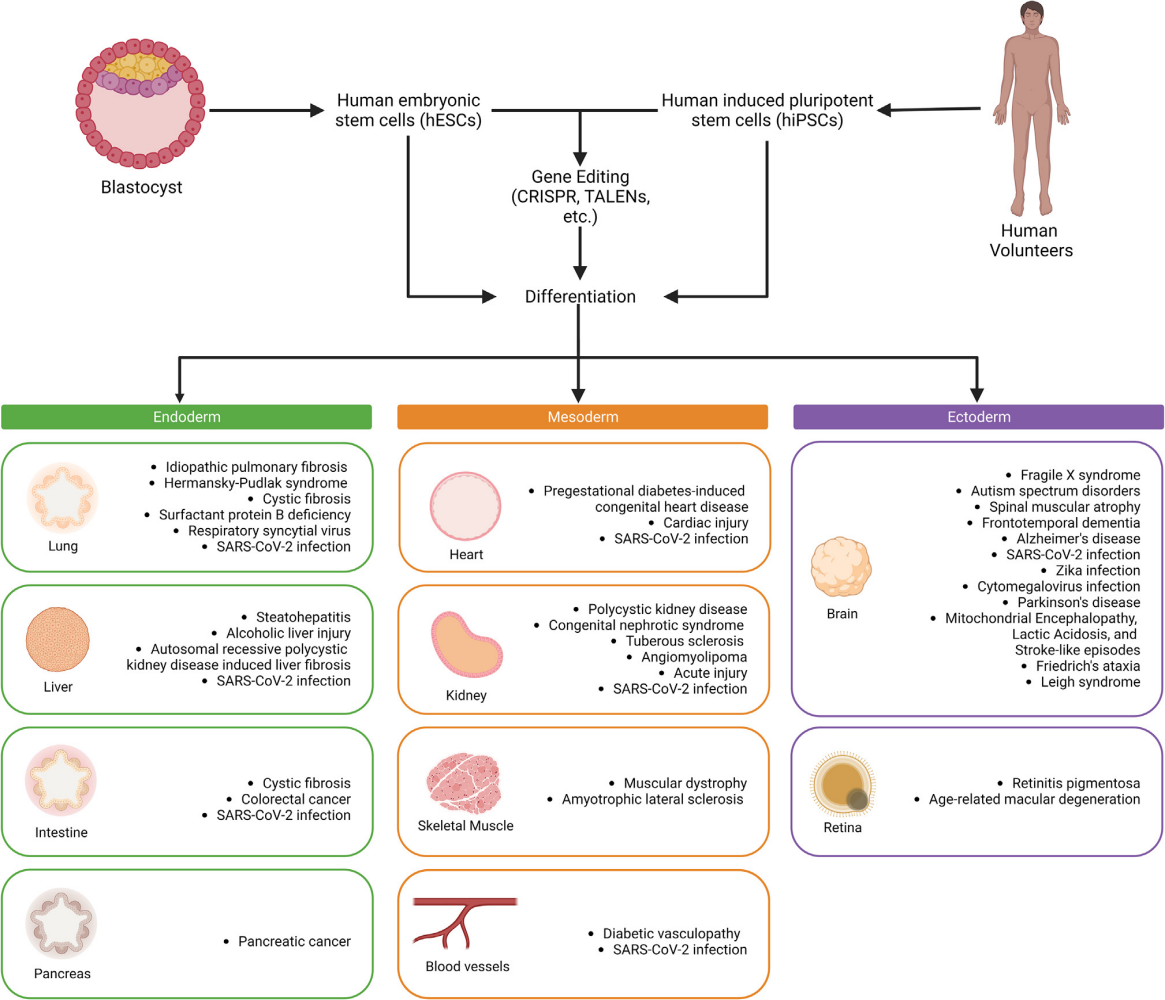
Strategies for using hPSC-derived organoids in disease modeling

Since their discovery, there have been significant efforts to apply hPSCs for disease modeling and drug discovery. iPSCs were derived from diseased patients and healthy individuals to model the cellular phenotypes involved in disease progression. Isogenic hPSCs were used for studying the impact of a single gene, regulatory region, or genetic variants (*single*-nucleotide polymorphisms [SNPs)) on cell fate^4^ (Figure 1). These models have served extensively as drug-discovery platforms and predictive indicators of drug response, leading to the development of unique therapeutic candidates and paving the way for the development of precision medicine.

Most early-stage hPSC-based disease-modeling work focused on two-dimensional (2D) cell cultures. Although 2D cultures are relatively easy to culture and scalable for high-throughput, high-content screens, they are monotypic and lack the diverse cell types and proper cell-cell interactions of 3D architecture. In the last decade, significant progress has been made in constructing 3D organoids that more faithfully resemble the organs of interest in terms of gene expression, protein expression, and cellular architecture. Although the definition of organoids varies, they are generally 3D structures derived from stem cells, progenitor cells, or differentiated cells that self-organize to recapitulate native tissue *in vitro.*^5^ These structures contain multiple cell types in an organized fashion, providing a more physiologically relevant context than 2D cell cultures. Moreover, organoids are also readily scalable, making them appropriate systems for high-throughput studies for drug discovery. In this review, we will summarize the current progress in using hPSC-derived organoids for drug discovery and genetic screening, as outlined in Table 1. Moreover, we discuss the potential applications of hPSC-derived organoids for precision medicine, as outlined in Table 2.

**Table 1:** hPSC-derived organoids used in chemical and genetic screens

Germ layer	Tissue	Disease modeled	Mutation(s) studied	Stem cell type	Model	Phenotype observed	Reference
Endoderm	lung	cystic fibrosis (CF)	*CFTR*	iPSC	proximal airway organoids	reduced forskolin-induced swelling	McCauley et al.^6^
		Hermansky-Pudlak syndrome (HPS)	*HPS*	ESC	lung organoids	pulmonary fibrosis	Strikoudis et al.^7^
		inherited deficiency of surfactant protein B	*SFTPB*	iPSC	lung organoids	abnormal lamellar body formation	Leibel et al.^8^
	liver	autosomal-recessive polycystic kidney disease (ARPKD)	*PKHD1*	iPSC	lung organoids	abnormal bile ducts and fibrosis	Guan et al.^9^
	intestinal	colorectal cancer associated with familial adenomatous polyposis (FAP)	*APC*	iPSC	colonic organoids	enhanced *WNT* activity and increased cell proliferation	Crespo et al.^10^
		cystic fibrosis	*CFTR*	iPSC	intestinal organoids	smaller steady-state lumen area and reduced forskolin-induced swelling	Mithal et al.^11^
	pancreas	pancreatic ductal adenocarcinoma	*TP53*	ESC	exocrine progenitor organoids	mortality *in vivo* after transplantation	Huang et al.^12^
			*KRAS*	iPSC, ESC	pancreatic duct-like organoids	heterogeneous dysplastic lesions or cancer	Breunig et al.^13^
			*KRAS* and *CDKN2A*	iPSC, ESC	pancreatic duct-like organoids	dedifferentiated pancreatic ductal adenocarcinomas	
			*GNAS*	iPSC, ESC	pancreatic duct-like organoids	intraductal papillary mucinous neoplasia-like structures	
			*GNAS*	iPSC, ESC	acinar organoids	acinar-to-ductal metaplasia-like changes	Huang et al.^14^
Mesoderm	kidney	angiomyolipoma (AML)	*TSC2*	iPSC	renal organoids	development of myomelanocytic cells and epithelial cysts	Hiratsuka et al.^15^
		autosomal-recessive polycystic kidney disease (ARPKD)	*PKHD1*	iPSC	organoid-on-a-chip	distal nephron cyst formation	Hiratsuka et al.^15^
		nephrotic syndrome	*NPHS1*	iPSC	kidney organoids	hypertrophied podocyte bodies	Hale et al.^16^
			*NOS1AP*	iPSC	kidney organoids	decreased tuft formation and increased apoptosis	Majmundar et al.^17^
		polycystic kidney disease	*PDK1* and *PDK2*	ESC	nephron-like kidney organoids	cyst formation	Tran et al.^18^
			*KIF3A* and *KIF3B*	iPSC, ESC	kidney organoids	lack of cilia, constitutive release of extracellular vesicles with signaling molecules associated with ciliopathy phenotypes	Cruz et al.^19^
		tuberous sclerosis	*TSC1* and *TSC2*	iPSC, ESC	renal organoids	angiomyolipoma, myomelanocytic phenotype	Pietrobon et al.^20^
	skeletal muscle	amyotrophic lateral sclerosis	*TDP-43*	iPSC	motor-neuron spheroids on a chip	decreased muscle contractions, degradation of motor neurons, and increased apoptosis in muscles	Osaki et al.^21^
		muscular dystrophies	*LMNA*	iPSC	multilineage muscle	nuclear elongation	Maffioletti et al.^22^
Ectoderm	neural	Alzheimer’s disease (AD)	*APOE*	iPSC	cerebral organoids	accelerated neural differentiation, reduced progenitor cell renewal, and impaired function of REST	Meyer et al.^23^
						exacerbated tau pathology	Zhao et al.^24^
					
		fragile X syndrome (FXS)	*FMRP*	iPSC	forebrain organoids	dysregulated neuronal development, maturation, and excitability	Kang et al.^25^
		Friedreich’s ataxia (FRDA)	*FXN*	iPSC	dorsal-root-ganglia organoids	impairment in axonal spreading	Mazzara et al.^26^
		frontotemporal dementia (FTD)	*MAPT*	iPSC	cerebral organoids	increased susceptibility to glutamate toxicity	Bowles et al.^27^
		Leigh syndrome (LS)	*SURF1*	iPSC	cortical brain organoids	compromised neural morphogenesis and retention of glycolytic proliferative state	Inak et al.^28^
		mitochondrial encephalomyopathy, lactic acidosis, and stroke-like episodes (MELAS)	*MTTL1*	iPSC	spinal-cord organoids	failure to differentiate into motor neurons	Khong et al.^29^
		Parkinson’s disease	*DNAJC6*	ESC	midbrain organoids	impaired midbrain-type dopamine neuron development via impaired WNT-LMX1A signaling	Wulansari et al.^30^
		spinal muscular atrophy	*SMN1*	iPSC	spinal organoids	motor-neuron degeneration	Hor et al.^31^
	retinal	retinitis pigmentosa (RP)	*RPGR*	iPSC	retinal epithelium and organoids	decreased photoreceptor number and cilial length	Deng et al.^32^
			*RP2*	iPSC	retinal organoids	photoreceptor degeneration	Lane et al.^33^

**Table 2:** Applications of hPSC-derived organoids in drug discovery and precision medicine

Tissue	Disease modeled	Cell types analyzed	Technique	Format	Assay	Major findings	Reference
Lung	SARS-CoV-2	hPSC-derived airway organoids	drug and/or chemical screening	384-well plates	immunofluorescence staining	GW6471, Xanthumol, 5-(tetradecyloxy-)2-furoic acid and ND-646 blocking SARS-CoV-2 infection.	Duan et al.^34^
	SARS-CoV-2	hPSC-derived lung organoids	drug and/or chemical screening	384-well plates	luciferase activity	imatinib, MPA and QNHC blocking SARS-CoV-2 entry	Han et al.^35^
	SARS-CoV-2	iPSC-derived lung organoids	drug and/or chemical screening	96-well plates	FRET protease activity assay	2-phenyl-1,2-benzoselenazol-3-one compounds inhibiting M^pro^; Lead E24 blocking viral replication.	Huff et al.^36^
Liver	ARPKD-associated liver fibrosis	iPSC-derived hepatic organoids	drug and/or chemical evaluation	96-well plates	immunostaining and mRNA assessment	imatinib, crenolanib, and sunitinib inhibiting ARPKD fibrosis	Guan et al.^9^
Intestinal	colorectal cancer	FAP-iPSC-derived colonic organoids	drug and/or chemical evaluation	3D spheroids in Matrigel droplets	immunoblot, immunostaining, RT-qPCR	geneticin rescuing APC expression, decrease WNT activity; and block hyperproliferation in the FAP-iPSC-derived colonic organoids	Crespo et al.^10^
Heart	cardiac injury	hPSC-derived cardiac organoids	drug and/or chemical screening	Heart-dyno culture inserts	high-content imaging, high throughput proteomics	activation of the mevalonate pathway was found to promote cardiac cell proliferation	Mills et al.^37^
	SARS-CoV-2	hPSC-derived cardiac organoids with endothelial cells	drug and/or chemical screening	collagen/Matrigel mix within inserts	force analysis	BET inhibitors, including apabetalone, rescue cardiomyocytes from SARS-CoV-2 infection	Mills et al.^37^
Kidney	polycystic kidney disease	hPSC-derived kidney organoids	drug and/or chemical screening	methylcellulose	live imaging	UCN-01, UCN-02, IKK inhibitor VII, and QNZ, inhibiting cyst formation	Tran et al.^18^
		Polycystic-kidney hPSC-derived kidney organoids	drug and/or chemical screening	96-well microwell plates	imaging	blebbistatin as a specific activator of cystogenesis	Czerniecki et al.^38^
		acute-kidney-injury hPSC-derived kidney organoids	drug and/or chemical evaluation	96-well ultralow adhesion plates	immunofluorescence	DNA ligase inhibitors such as the DNA ligase IV inhibitor SCR7 rescuing tubular injury	Gupta et al.^39^
		polycystic kidney hPSC-derived disease kidney organoids	drug and/or chemical evaluation	multifluidic chip	3D imaging by tissue clearing of organoids	R-naproxen and T-5224 inhibiting cyst formation.	Hiratsuka et al.^15^
	chronic kidney disease and ciliopathies	iPSC-derived kidney organoids	genetic screening	96-well round bottom plates	RNA-seq, immunofluorescence, pooled CRISPR screening	monogenetic and complex trait kidney disease genes and a large candidate list of ciliopathy-related genes	Ungricht et al.^40^
	nephrotic syndrome	iPSC-derived kidney organoids	genetic screening	transwell inserts	immunofluorescence and light microscopy	impaired glomerulogenesis and impaired podocytes.	Majmundar et al.^17^
	TSC/AML	iPSC-derived kidney organoids	drug and/or chemical evaluation	transplanted to the kidney capsule *in vivo*	immunofluorescence, immunoblot, *in vivo* studies	rapamycin-loaded nanoparticles were found to ablate *TSC2*^*−/−*^ AML organoid xenografts	Hernandez et al.^41^
	Kidney diseases	iPSC-derived kidney organoids	drug and/or chemical evaluation	transplanted to the kidney capsule *in vivo*	single-cell RNA-seq, electrophysiology, immunofluorescence.	GFB-887 displayed significant protection of kidney filter cells in a rat model transplanted with human kidney organoids	Westerling-Bui et al.^42^
	SARS-CoV-2	iPSC-derived kidney organoids	drug and/or chemical evaluation	3D kidney organoids grown as cell aggregates at an air-medium interface.	RT-qPCR, Plaque assays	MAT-POS-b3e365b9-1, prevented viral replication in kidney organoids.	Jansen et al.^43^
Skeletal muscle	ALS	hPSC-derived skeletal muscle cells and motor neuron spheroids	drug and/or chemical evaluation	ALS-on-a-chip technology	immunofluorescence and electrical and chemical stimulation	muscle contractions in the ALS motor unit could be recovered by single treatments with rapamycin and co-treatments with rapamycin and bosutinib	Osaki et al.^21^
Neural	Alzheimer’s disease	iPSC-derived cerebral organoids	genetic screening	10-cm plates on an orbital shaker	RNA-seq	impaired RNA metabolism and an increase in the number of stress granules	Zhao et al.^24^
	frontotemporal dementia	iPSC-derived cerebral organoids	drug and/or chemical evaluation	6-well low-attachment plates	longitudinal imaging to track survival	glutamatergic toxicity in tau-V337M organoids can be reversed by apilimod, a PIKFYVE inhibitor	Bowles et al.^27^
	SMA	iPSC-derived SMA organoids	drug and/or chemical evaluation	Low-attachment plate	immunofluorescence	PD 0332991 prevents motor degeneration	Hor et al.^31^
	MELAS	iPSC-derived spinal cord organoids	drug and/or chemical evaluation	embedded in matrigel droplets	immunofluorescence and qPCR	DAPT overcomes neurodevelopmental defects in MELAS organoids	Khong et al.^29^
	fragile X syndrome	FXS-iPSC-derived human forebrain organoids	drug and/or chemical evaluation	12-well spinning bioreactors	immunofluorescence	PI3K inhibitors such as LY294002 and GSK2636771 rescue developmental deficits	Kang et al.^25^
	Zika	hPSC-derived forebrain organoids	drug and/or chemical screening	low-adhesion V-bottom 96-well plates	immunofluorescence	hippeastrine inhibits ZIKV infection in forebrain organoids and reverse developmental defects	Zhou et al.^44^
	endocrine toxicity	iPSC-derived cortical organoids	drug and/or chemical screening	low attachment plate	urinalysis, RNA-seq, immunofluorescence	endocrine disrupting mixtures disrupt hormone-regulated and disease-relevant neurodevelopment	Caporale et al.^45^
	effects of narcotic and neuropsychiatric factors on development	iPSC-derived dorsal forebrain organoids	drug screening	Low-attachment Aggrewell V-bottom plates	BrdU pulse-chase assay, immunofluorescence, proteomics, FACs	exposure to cannabinoids increases apoptosis and DNA damage, causing neurotoxicity	Notaras et al.^46^
Eye	age-related macular degeneration	iPSC-derived retinal organoids	drug and/or chemical evaluation	manually isolated retinal epithelial domains	immunofluorescence	PIEZO1 activators, such as YODA1, induce photoreceptor extrusion, thus reversing photoreceptor neurodegeneration	Völkner et al.^47^

### Endoderm derivatives

#### Lung organoids

The lungs are a vital pair of spongy, air-filled organs that enable respiration. The trachea conducts inhaled air into the lungs through its tubular branches, called bronchi. These bronchi divide into smaller branches known as bronchioles, which eventually end in clusters of microscopic air sacs called alveoli. Researchers have developed several protocols to direct the stepwise differentiation of hPSCs toward different types of lung organoids, including airway organoids,^6^ alveoli organoids,^48^ alveoli type 2 cells,^49^ and lung organoids.^50^^,^^51^^,^^52^

Many studies have used hPSC-derived airway and alveolar organoids to investigate molecular targets and perform drug evaluation. For example, isogenic hPSCs carrying CRISPR/Cas9-mediated knock-out mutations of genes associated with the Hermansky-Pudlak syndrome (HPS) were employed in a study of the underlying pathogenesis of idiopathic pulmonary fibrosis (IPF) because HPS patients develop HPS-associated interstitial pneumonia (HPSIP), which resembles IPF.^7^ In this study, *HPS1*^*−/−*^, *HPS2*^*−/−*^*,* and *HPS4*^*-/*^,^*-*^ but not *HPS8*^*−/−*^ hPSC-derived lung organoids displayed fibrotic features, such as the accumulation of mesenchymal cells, and the enhanced deposition of collagens and fibronectin, which correlated with the clinical incidence of HPSIP. Transcriptome analysis of purified epithelial cells identified the upregulation of interleukin-11 (IL-11) as a potential inducer of fibrosis in the mutant organoids, suggesting IL-11 as a potential molecular target for HPSIP .

In addition, iPSC-derived airway organoids have been used for studying cystic fibrosis, a disease caused by mutations in *CFTR*. iPSCs derived from patients with cystic fibrosis due to homozygous ΔF508 mutations were used for evaluating CFTR-dependent organoid swelling in response to forskolin,^6^ a CFTR activator. The organoids derived from CF-iPSCs lose their responsiveness to forskolin, but zinc-finger nuclease (ZFN)-based gene editing correcting the mutations restored the responsiveness. Moreover, *SFTPB* ^*Pro133GlnfsTer95*^ iPSC-derived lung organoids were used for modeling Surfactant protein B (SFTPB) deficiency, a fatal disease affecting infants. The *SFTPB*^*Pro133GlnfsTer95*^ iPSC-derived lung organoids showed abnormal lamellar-body formation and no tubular myelin. This was reversed by infection with a lentivirus carrying *SFTPB*, resulting in an increase in secreted dipalmitoyl phosphotidylcholine (DPPC) and the restoration of normal lamellar bodies.^8^ Therefore, iPSC-derived lung organoids could serve as a useful platform for testing therapeutic approaches, such as gene delivery.

In addition to inherited diseases, hPSC-derived lung organoids are also being used in studies of viral infection. Previous studies have shown that respiratory syncytial virus (RSV) infection of lung bud organoids, which can develop into both airway and alveolar structures, leads to swelling, detachment, and shedding of infected cells into the organoid lumen.^9^ Such platforms are crucial because there is no model that accurately mimics human RSV infection, and there are currently no available vaccine or antiviral therapy for RSV. Lung organoids have also been useful in understanding SARS-CoV-2 infection because it significantly affects the respiratory tract. Differentiated lung alveolar organoids are enriched in epithelial cells, including alveolar type 2 (AT-2) cells, which are the main sites of infection. These AT-2 cells express SARS-CoV-2 entry factors, including angiotensin-converting enzyme 2 (ACE2) and TMPRSS2. During the COVID-19 outbreak, hPSC-derived lung organoids have been extensively used for the discovery of novel drugs for SARS-CoV-2. The first high-throughput screen using hPSC-derived lung organoids (hPSC-LOs) identified several inhibitors blocking the entry of SARS-CoV-2; such inhibitors include imatinib, mycophenolic acid (MPA), and quinacrine dihydrochloride (QNHC).^35^ hPSO-LOs were first treated with a library of drugs approved by the FDA and then infected with a vesicular stomatitis ΔG-luciferase pseudotyped with the SARS-CoV-2 spike protein. Imatinib and QNHC were both found to have a potential binding to ACE2, and QNHC and MPA treatment reduced FURIN expression. Moreover, imatinib was found to transform fatty acid biosynthesis and metabolism. Additionally, antiandrogenic agents have been shown to lower ACE2 expression, highlighting a novel crosstalk between androgen and the expression of viral receptors and co-receptors.^53^ A class of 2-phenyl-1,2-benzoselenazol-3-one compounds with improved physicochemical properties was designed via rational drug design and *in silico* docking with the parent compound ebselen as a basis for targeting the SARS-CoV-2 main protease. Once again, iPSC-derived lung organoids were pre-treated with a 2-phenyl-1,2-benzoselenazol-3-one compound prior to infection, leading to severe depletion of viral RNA levels, demonstrating the successful inhibition of viral replication.^36^ hPSC-derived airway organoids (hPSC-AOs) that accurately recapitulate viral-host interactions have been developed. For instance, cytokine and chemokine signaling is upregulated in infected organoids, similar to the inflammatory changes observed in primary human infections. A high-content screen was performed with hPSC-AOs via an FDA-approved drug library in a 384-well format. hPSC-AOs were pre-treated with the compounds before SARS-CoV-2 infection. The organoids were then fixed and stained so the percentage of SARS-N^+^ cells could be determined. The compound GW6471 was identified as being able to block SARS-CoV-2 infection by inhibiting the hypoxia-inducible factor 1 subunit alpha-glycolysis axis and fatty acid synthesis and metabolism.^34^ In addition to GW66471, three other compounds were also observed to be effective in blocking SARS-CoV-2 in these organoids: xanthohumol, 5-(tetradecyloxy)-2-furoic acid, and ND-646, all of which inhibit fatty acid synthesis.

#### Liver organoids

The liver is the largest solid organ in the body and plays vital roles in removing waste products and foreign substances from the bloodstream, regulating blood sugar levels, and creating essential nutrients. Many protocols have been reported to direct the differentiation of hPSCs toward liver cells or liver organoids consisting of hepatocyte-, stellate-, and Kupffer-like cells.^54^^,^^55^^,^^56^^,^^57^^,^^58^^,^^59^ These hPSC-derived liver organoids have been applied to the study of complex pathologies that cannot be recapitulated by simpler 2D systems. For instance, iPSC lines from healthy and steatohepatitis patients were differentiated into multicellular liver organoids in a study of steatohepatitis mechanisms.^60^ The resulting steatohepatitis iPSC-derived liver organoids displayed steatosis and fibrosis phenotypes upon free fatty acid treatment, which could not be observed in simpler reaggregated co-cultured spheroids. In addition, iPSC-derived liver organoids of the patients with genetic lysosomal enzyme deficiency showed that the pathological features of steatohepatitis could be rescued via farnesoid X receptor agonism with obeticholic acid^61^ and FGF19, which block lipid accumulation and reduce reactive oxidative species (ROS) levels. Moreover, the stiffness of such hPSC-derived liver organoids was correlated with the severity of fibrosis, and a stiffness increase per organoid was observed via atomic-force microscopy^24^ in severe fibrosis. Organoids provide a simple *in vitro* platform for such parameters to be measured via suitable assays, similar to standard clinical practices of measuring liver stiffness in non-alcoholic steatohepatitis (NASH) patients via non-invasive elastography. Furthermore, a co-culture system of hESC-derived hepatic organoids (hEHOs) and human fetal liver mesenchymal cells was used for studying alcoholic liver disease.^60^ The hEHOs generated in this study were restricted to hepatocyte and cholangiocyte lineages and were scalable; they exhibited expandability for up to 20 passages. Additionally, the hEHOs could be established from just a single dissociated hEHO cell, overcoming limitations associated with the availability and throughput of human primary liver tissues. Currently, there are no FDA-approved drugs for alcoholic liver disease (ALD), and the hEHOs can serve as a suitable model for drug discovery in ALD. Upon ethanol treatment, the hEHOs showed increased oxidative stress generation, steatosis, release of inflammatory mediators, and fibrosis. Interleukin-1 (IL-1) and interleukin-17 (IL-17) signaling were specifically increased after ethanol treatment, and these signaling pathways could be druggable targets in ALD. iPSC-derived hepatic organoids were used for studying autosomal-recessive polycystic kidney disease (ARPKD), a disease in which patients who survive the perinatal period experience severe forms of liver disease.^9^ These hepatic organoids exhibiting the key characteristics of ARPKD liver pathology can be developed in just 21 days. Moreover, in contrast to other model systems, the iPSC-derived hepatic organoids recapitulate the 3D architecture of the liver, possess the cell types necessary for fibrogenesis, and do not require injury-induced activation, which leads to experimental heterogeneity. Hepatic organoids derived from isogenic hPSC lines carrying the causative mutation in *PKHD1* display abnormal bile ducts, fibrosis, collagen abundance, and thick collagen fiber production. The fibrotic effect in *PKHD1* mutant iPSC-derived hepatic organoids was reversed through the use of PDGFRB inhibitors such as crenolanib, sunitinib, and imatinib, which ameliorated collagen formation in ARPKD organoids and reduced collagen and hydroxyproline, providing treatment options that potentially could be used in liver fibrosis. More recently, human fetal hepatocyte organoids were used for modeling free fatty acid loading, interindividual genetic variability (PNPLA3 I148M), monogenic lipid disorders (*APOB* and *MTTP* mutations), and induced non-alcoholic fatty liver disease (NAFLD). Chemical screening using human fetal hepatocyte organoids revealed compounds that were effective at resolving steatosis.^62^ Finally, hPSC-derived liver organoids can recapitulate infectious diseases, such as SARS-CoV-2 infection. SARS-CoV-2 infection significantly upregulated inflammatory pathways, cytokine-cytokine receptor interaction, and IL-17 signaling in both infected hepatocyte organoids and cholangiocyte organoids, providing insights into targeting pathways for SARS-CoV-2 infection in the liver.^63^

#### Intestine organoids

The first stepwise protocol to differentiate hPSCs into multicellular intestinal organoids was reported in 2011.^64^ Soon after, strategies were reported for deriving colon organoids.^64^^,^^65^ iPSCs derived from patients with familial adenomatous polyposis (FAP) and carrying mutations in *APC* have been used for modeling colorectal cancer and evaluating drugs that reverse FAP.^10^ FAP-iPSC-derived colonic organoids displayed increased epithelial cell proliferation, and compounds known to reverse the tumor-like properties in colorectal cancer were investigated. Geneticin, a ribosome-binding compound, was found to specifically decrease the hyperproliferation in FAP-iPSC-derived colonic organoids by inhibiting the recognition of the premature stop codon in *APC* by the ribosome and thus to restore *APC* function. Additionally, *CFTR*^*Δ508*^ iPSC-derived mesenchyme-free human intestinal organoids (HIOs) have successfully modeled cystic fibrosis; these HIOs exhibit a smaller lumen area and defective forskolin-induced swelling when compared to WT iPSC-derived HIOs. These defects were rescued in corrected HIOs, demonstrating the potential of HIOs to aid in evaluation of new approaches to gene therapy.^11^ Primary rectal organoids are another system used for evaluating CFTR function and can accurately distinguish responders from non-responders *in vivo.*^66^ Although the development of hPSC-derived HIOs is time-consuming and labor-intensive in comparison to that of primary organoids, they are more scalable and can overcome genetic variations arising with the use of primary tissues. More recently, hPSC-derived colonic organoids have been applied to the study of viral infection. It is observed that SARS-CoV-2 could infect hPSC-derived colon organoids and mimic the gastrointestinal symptoms of COVID-19 patients. Drugs such as imatinib, MPA, and QNHC, which block viral entry in lung organoids, were found to also inhibit virus infection in colon organoids.^35^

#### Pancreas

The pancreas is a major gland that plays essential roles in both the digestive and endocrine systems. Over the last two decades, significant efforts have been made to develop protocols for differentiating hPSCs into pancreatic endocrine and exocrine cells and organoids.^67^^,^^68^

One study used hPSC-derived pancreatic organoids to model pancreatic ductal adenocarcinoma (PDAC), which is believed to originate in the exocrine and ductal compartment. TP53^R175H^ was found to induce cytosolic SOX9 localization in hPSC-derived exocrine progenitor spheroids, which can branch into acinar and ductal structures, causing mortality after transplantation *in vivo.*^12^ Few *in vitro* models of the exocrine pancreas exist, making the development of hPSC-derived exocrine pancreatic organoids an exciting avenue for drug discovery for PDAC. In addition, hPSC-derived pancreatic duct-like organoids (PDLOs) successfully recapitulated pancreatic dysplasia *in vitro* and carcinogenesis *in vivo*.^13^ The PDLOs were derived from hPSCs overexpressing oncogenic mutant *GNAS, KRAS,* or *KRAS* together with *CDKN2A* knockout, as well as iPSCs from a McCune-Albright patient. After *in vivo* transplantation, PDLOs with oncogenic *KRAS* form heterogeneous dysplastic lesions, PDLOs carrying oncogenic *KRAS* with *CDKN2A* knockout give rise to pancreatic ductal adenocarcinomas, and PDLOs carrying oncogenic *GNAS* form intraductal papillary mucinous neoplasia-like structures. Therefore, the development of PDLOs enabled the investigation of cancer progression and dysplasia in a system with a defined genetic background, allowing the tracing of the origins of cancer as well as an investigation of intermediate states. Such longitudinal studies along the disease spectrum can facilitate the discovery of drugs that are most appropriate for each stage of disease progression. Moreover, the genetic background of the starting hPSCs in organoids can be controlled, and such cells are amenable to gene editing. In another similar study, the overexpression of *GNAS*^*R201C*^ induced cystic growth in hPSC-derived ductal organoids, and the overexpression of *KRAS*^*G12D*^ in acinar organoids induced acinar-to-ductal metaplasia-like changes, exhibiting lineage-specific tropism in response to the induction of oncogenic mutations.^14^

### Mesoderm derivatives

#### Heart organoids

As one of the first cell types derived from hPSCs in monolayer culture, hPSC-derived cardiomyocytes have been extensively studied for their potential in modeling congenital or familial heart defects^69^ and aiding in the assessment of drug-induced cardiac toxicity.^70^ Congenital heart defects,^71^ which often involve structural defects, are the most common type of congenital defect in humans and require 3D organoids for accurate modeling. A recent study used human heart organoids to model pregestational diabetes (PGD)-induced CHD.^72^ The heart organoids exhibited well-defined internal chambers containing multiple cardiac cell types and a complex vasculature and demonstrated enhanced functionality. Culturing the human heart organoids in PGD conditions resulted in cardiac hypertrophy, inappropriate patterning, reduced mitochondria, abnormal lipid metabolism, and impaired organization. This suggests that the complex heart organoids could be useful models that would help with identifying pharmacological agents that might reverse this condition. Drug screening using hPSC-cardiac organoids in a 96-well format identified compounds that have the potential to promote proliferation without adverse side effects on contractility.^73^ This study evaluated 105 small molecules with regenerative potential and identified two compounds that promote proliferation via distinct mechanisms involving MAPK14 signaling and regulation of an extracellular matrix and cholesterol biosynthesis networks. Surprisingly, many hits identified in 2D screening turned out to be ineffective in the 3D cardiac organoids or caused adverse effects on cardiac contractility. Many drugs identified for cardiac pathologies via 2D cell culture systems and animal models are not translatable in humans because of immaturity of the existing models and inter-species specific differences. Thus, drug screening using complex human cardiac organoid models can potentially reveal more promising drug candidates and fulfill the unmet need for therapeutics required for cardiac diseases.

Furthermore, hPSC-derived organoids have been used for studying viral infection, including COVID-19 infection. Although respiratory symptoms are the typical manifestation of COVID-19, cardiac symptoms, such as myocardial injury, are also common. Mills et al. used hPSC-derived cardiac organoids to screen for drugs that prevent cytokine-storm- and/or viral-infection-induced cardiac dysfunction.^37^ They utilized human cardiac organoids co-cultured with endothelial cells, forming branched endothelial structures surrounded by pericytes, which presented a more suitable model system that retains microenvironment interactions. They employed a combination of human cardiac organoids with tools such as phosphoproteomics and single-nucleus RNA sequencing to uncover novel molecular targets and pathways to reverse SARS-CoV-2 infection. Bromodomain and extra-terminal motif family (BET) inhibition was identified as a key mechanism for alleviating the effects of the cytokine storm, and BD-2 selective compounds RXV-2157 and apabetalone demonstrated efficacy in this study. Apabetalone reduced diastolic dysfunction, downregulated ACE2, and decreased viral infection.

#### Kidney organoids

Chronic kidney disease affects 15% of the adult population in the United States, making it a significant healthcare burden. Polycystic kidney disease, which involves the typical pathological cystic expansion of tubular epithelial cells, has been extensively modeled with hPSC-derived kidney organoids. The tubular cells of nephron-like kidney organoids derived from hPSCs carrying biallelic loss-of-function mutations in *PKD1* or *PKD2* display cyst formation.^18^ Cystic outgrowths could be maintained for months in culture, and tens of thousands of homogeneous kidney organoids could be generated at one time. Screening of 247 protein kinase inhibitors in a live imaging assay identified UCN-01, UCN-02, I κ B kinase (IKK) inhibitor VII, and quinazoline as inhibiting cyst formation in *PKD1*^*−/−*^ and *PKD2*^*−/−*^ kidney organoids without adversely affecting organoid growth. This is particularly beneficial given the limited availability of pharmacological interventions for autosomal-dominant polycystic kidney disease (ADPKD) patients. Currently, only one FDA-approved drug, tolvaptan, is available, and this drug inhibits disease progression in only a subset of ADPKD patients. In another study, *KIF3A*^*−/−*^ and *KIF3B*^*−/-*^ hPSC-derived organoids were used for studying conditions along the ciliopathy spectrum, including defective neurogenesis, impaired nephrogenesis, and polycystic kidney disease.^19^ Animal models cannot be adapted to the investigation of ciliary phenotypes because the inhibition of intraflagellar transport results in ciliary ablation and embryonic lethality. Automation has been achieved in the generation of kidney organoids in just 21 days in 96-well- and 384-well-plate formats. Additionally, VEGF supplementation was used to increase the generation of endothelial cells (ECs) to provide a suitable microenvironment for the kidney organoids. Automated multidimensional phenotyping involving the use of liquid robots for all the steps of plating, differentiation, fixation, and phenotyping identified blebbistatin, an inhibitor of NMII ATPase activity, as an activator of autosomal-dominant polycystic kidney disease cystogenesis. This indicates that polycystins might regulate actomyosin activation within the tubular epithelium of stromal myofibroblasts to prevent cyst formation.^38^

Automated multidimensional phenotyping could be applied for high-throughput drug discovery, reducing the labor limitations and heterogeneity associated with time-consuming differentiation steps and processing for immunofluorescence or other assays aimed at assessing desired phenotypes. ARPKD patients develop enlarged kidneys and show a progressive loss in renal function, often resulting in dialysis and kidney transplantation. A physiologically relevant organ-on-a-chip model utilizing *PKHD1*^*−/−*^ organoids detected mechanosensing signals as the basis of cystogenesis.^15^ Such organ-on-a-chip models might provide a suitable dynamic cellular microenvironment by introducing fluidic flow, making them more representative models that can be used for drug discovery and validation. Additionally, in the case of ARPKD, cystogenesis has not been observed in static organoid models without external stimuli, such as treatment with cAMP. The *PKHD1*^*−/−*^ hPSC-derived organoids generated in this platform exhibited cytoskeletal remodeling, negative cell-cycle regulation, and RAC1 and FOS upregulation. The FDA-approved nonsteroidal anti-inflammatory drugs (NSAIDs) R-naproxen, R-ketorolac, and T-5224 were shown to effectively inhibit cyst growth via the downregulation of RAC1 and FOS signaling.

Besides PKD, hPSC-derived kidney organoids are used for studying congenital nephrotic syndrome. Defects in the globular basement membrane (GBM), loss of slit diaphragms, and destruction of the foot process are underlying causes of congenital nephrotic syndrome. The intact glomeruli from hiPSC kidney organoids have been isolated and termed OrgGloms.^16^ OrgGloms from congenital nephrotic syndrome patients with the exon 10 and exon 27 *NPHS1* variants exhibited reductions in nephrin and podocin. Patients homozygous for *NPHS1* mutation displayed a complete lack of nephrin, slightly upregulated levels of podocin, and decreased NEPH1 levels. Such systems might be appropriate for the drug discovery for human glomerular diseases. The introduction of the recessive *NOS1AP* variants into iPSCs revealed decreased tuft formation, increased pyknosis, and apoptosis in iPSC-derived kidney organoids.^17^ In contrast, endogenous *NOS1AP* was not detectable in 2D podocyte culture systems. Additionally, *TSC1*^*−/−*^ and *TSC2*^*−/−*^ hPSC-derived renal organoids model the tuberous sclerosis complex, including multiple developmental defects in the epithelial, stromal, and glial compartments.^20^ *TSC2*^*−/−*^ hiPSC-derived renal organoids successfully create renal angiomyolipoma (AML) organoids, which share a transcriptional signature and myomelanocytic phenotype with physiological AML and develop epithelial cysts, recapitulating AML lesions.^41^ Transplantation of *TSC2*^*−/−*^ organoids into immunodeficient rats results in fully vascularized human AML xenografts that could be used to test a therapeutic approach involving the use of nanocarriers to increase rapalog bioavailability at AML sites. During severe or repetitive injury, human kidney organoids develop fibrosis, and incomplete repair occurs because of *FANCD2* loss, resulting in a downregulation of homology-directed repair (HDR). In this context, human kidney organoids can be used for studying intermediate states during the transition from intrinsic to incomplete repair. Targeted drug screening identified SCR7 as a candidate for rescuing FANCD2- and/or RAD51-mediated repair in a cisplatin-induced organoid-injury model, restoring HDR and preventing chronic kidney disease.^39^ hPSC-derived kidney organoids also serve as valuable tools for pharmacodynamic^71^ studies. A recent study transplanted iPSC-derived kidney organoids into rats and performed PD studies of GFB-887, a novel investigational TRCP5 inhibitor, before its evaluation in a phase 2 clinical trial with patients with TRPC-mediated focal segmental glomerulosclerosis (FSGS).^42^ GFB-887 displayed significant protection of podocytes, which further strengthens the rationale for moving this drug into more advanced clinical studies.

Because many COVID-19 patients develop kidney symptoms, several studies have investigated the impact of SARS-CoV-2 infection by using hPSC-derived kidney organoids. SARS-CoV-2 infects podocytes and tubular epithelium, resulting in increased profibrotic signaling, cellular injury, and inflammatory responses. This can be reversed with the use of a protease blocker, MAT-POS-b3e365b9-1, which blocks viral replication in kidney organoids.^43^ Recent studies have shown that infected 3D kidney spheroids, which lack an interstitial compartment, do not exhibit fibrosis or associated injury molecules. This further demonstrates that kidney organoids, which display advanced epithelial-interstitial cellular complexity, serve as suitable systems for dissecting mechanistic processes and drug validation. Additionally, whole-genome pooled CRISPR screening, involving a dox-inducible Cas9 system in iPSC-derived kidney organoids, identified a long list of candidate genes implicated in ciliopathies and confirmed two congenital abnormalities involving the kidney and urinary-tract genes *CAKUT*, *CCDC170*, and *MYH7B*. Furthermore, a *cis*-inhibitory effect of Jagged1, which controls epithelial proliferation, was found to be necessary for inhibiting cystic growth.^40^ Spatiotemporal induction of Cas9 enabled longitudinal sampling of cells along different lineages, including tubular, stromal, or podocyte cell types. These findings demonstrate the tractability of such systems in drug discovery and disease modeling.

#### Skeletal-muscle organoids

Researchers have successfully modeled a range of muscular dystrophies, such as Duchenne, limb-girdle, and lamin A/C (*LMNA*)-related muscular dystrophies, by using 3D artificial skeletal muscle tissue derived from patient iPSCs.^22^ These iPSC-derived skeletal muscles, created within fibrin hydrogels under unidirectional tension, are multicellular and contain important cellular components such as endothelial cells, pericytes, and motor neurons. The fidelity of this platform was validated in *LMNA* iPSC-derived muscle tissues, which displayed nuclear elongation consistent with results seen in *LMNA*-mutant mice and primary myoblasts. Another promising application of these 3D system is in the study of amyotrophic lateral sclerosis,^72^ a fatal type of motor neuron disease with limited treatment options. One study applied an “ALS-on-a-chip” model containing iPSC-derived 3D skeletal muscle bundles and light-sensitive-channel rhodopsin-2-induced motor neuron spheroids to model ALS and screen for drug candidates.^21^ The model used light to activate muscle contraction, and results showed that compared to the non-ALS hESC-derived motor unit, the ALS motor unit displayed fewer muscle contractions, motor neuron degradation, and apoptosis. Co-treatment with rapamycin and bosutinib reversed these negative effects by upregulating autophagy and increasing degradation of the TAR DNA-binding protein-43 in the motor neurons. Overall, iPSC-derived 3D skeletal-muscle models provide an excellent platform for drug screening and validation for muscular dystrophies and motor-neuron diseases.

#### Blood-vessel organoids

Human blood-vessel organoids generated from hPSCs have been shown to comprise endothelial cells and pericytes arranged in capillary networks surrounded by a basement membrane. Suchorganoids have been used for modeling diseases such as diabetic vasculopathy, in which there is an expansion of the basement membrane and loss of vascular cells.^74^ Notably, using blood-vessel organoids to study diabetic vasculopathy has led to the discovery of novel disease mechanisms, including those involving *NOTCH3* and *DLL4*. In one study, diabetic mice transplanted with these human blood-vessel organoids were successfully treated with *NOTCH3* inhibitors, such as DAPT, which attenuated microvascular pathologies, thus demonstrating the potential of this platform for drug discovery targeting vascular diseases. Furthermore, iPSC-derived human capillary organoids express ACE2 at high levels, making them susceptible to SARS-CoV-2 infection. Treatment with human recombinant soluble ACE2 can protect these vascular organoids against infection.^75^ The early treatment of vascular organoids is especially critical because SARS-CoV-2 must first enter the bloodstream via the vascular endothelial cells before it can migrate to other organs, unless there is prior tissue damage. Therefore, using blood vessel organoids as a platform for drug discovery for SARS-CoV-2 is crucial for preventing multi-organ dysfunction in COVID-19 patients.

### Ectoderm derivatives

#### Neural organoids

##### Neurodegenerative disorders

Neurodegenerative disorders refer to a group of conditions that include Alzheimer’s disease (AD), Parkinson’s disease (PD), and other disorders characterized by accumulations of misfolded proteins and the loss of functional neurons in affected brain regions.^76^ AD is a neurodegenerative disease that causes dementia and other symptoms as a result of the abnormal formation of amyloid-β plaques. Among the apolipoprotein E (APOE) variants, APOE ε4 (*APOE4*) is the strongest genetic risk factor for AD. Studies have shown that iPSC-derived cerebral organoids bearing *APOE4* allele variants display an increased AD pathology, including elevated amyloid-β levels, apoptosis, and reduced synaptic integrity. In addition, *APOE4* most likely exacerbates p-tau accumulation, further contributing to the severity of AD pathology. Conversion of *APOE4* to *APOE3* in these organoids reduced the severity of AD pathology, indicating that *APOE4* is a promising molecular target for drug discovery of AD.^24^

Furthermore, hESC-derived cortical organoids containing microglia have been used for modeling AD-like inflammation and microglia function. These organoids exhibited significant reduction in amyloid--induced apoptosis, ferroptosis, and AD stage III in comparison to organoids lacking microglia. In a recent study, CRISPRi and CRISPR-droplet sequencing were performed so that the regulation of AD-linked genes in response to amyloid-β treatment could be investigated. Upon treatment with an amyloid-β DNA oligo that mimics AD-induced inflammation, *Trem2*-or S*orl1*-suppressed cortical organoids containing microglia showed significant reduction of cholesterol metabolic genes.^77^ The use of 3D organoids provides a more accurate and complex platform for studying AD pathology and offers the potential for new insights and therapeutic targets.

PD^71^ is characterized by the loss of dopamine neurons in the midbrain; this loss results in motor and non-motor symptoms. hESC-derived midbrain organoids that carry a loss-of-function mutation in *DNAJC6*, a gene associated with early-onset PD, have been shown to recapitulate PD pathology, exhibited defects in midbrain dopamine neuron development, and revealed *LMX1A* (LIM homeobox transcription factor 1 alpha) as a central factor in *DNAJC6*-mediated PD.^30^ This study was one of the first to provide evidence of a neurodevelopmental defect contributing to PD. In contrast, PD-associated phenotypes could not be recapitulated in *DNAJC6*-knockout mice, and primary human samples from PD patients are limited in availability. Thus, hPSC-derived midbrain organoids provide a valuable resource for the investigation of PD progression and drug discovery.

Frontotemporal dementia (FTD) is a heritable disease caused by *MAPT* mutations that result in the accumulation of tau and glutamatergic cortical neuronal death. iPSC-derived cerebral organoids expressing a *MAPT* mutation (tau-V337M) have been used to model early FTD pathogenesis; the resulting models include alterations in the expression of *MAPT* and *ELAVL4*, alterations in glutamatergic signaling, increased stress granules, and disruption of autophagy mechanisms. *MAPT* mutant organoids were susceptible to glutamate toxicity, but this phenotype was rescued by PIKFYVE inhibition via apilimod, illustrating the utility of brain organoids to identify potential therapeutics.^27^

Other neural organoids have been used for modeling neurodegenerative diseases. Friedreich’s ataxia (FRDA) is caused by the expansion of GAA repeats in the first intron of *FXN*, resulting in *FXN* silencing or reduced expression, consequently leading to mitochondrial dysfunction and defective iron metabolism. Dorsal-root-ganglia organoids derived from FRDA-iPSCs have demonstrated that removal of the entire first intron reduced pathology more than the removal of the GAA repeat tract alone, suggesting potential therapeutic targeted gene editing.^26^ Spinal muscular atrophy is a disease caused by *SMN1* mutations, resulting in decreased levels of SMN protein and subsequent death of spinal motor neurons and denervation of skeletal muscles. Spinal organoids derived from spinal-muscular-atrophy patient iPSCs identified high expression levels of cyclin-dependent kinases (CDKs) and cyclins in motor neurons, leading to degradation of motor neurons in these organoids. Chemical inhibition of CDK4/6 with palbociclib (PD 0332991) increased motor neuron survival, revealing a potential therapeutic target for spinal muscular atrophy.^31^

##### Neurodevelopmental disorders

Mitochondrial diseases can cause neurodevelopmental defects. Loss-of-function mutations in surfeit locus protein 1 (*SURF1*) results in a severe pediatric mitochondrial disease called Leigh syndrome (LS), for which there is no cure as of yet. LS-iPSC-derived brain organoids with mutations in *SURF1* showed compromised neural morphogenesis, and neural progenitor cells retained a proliferative glycolytic state as opposed to switching to OXPHOS, resulting in impaired neuronal generation and reduced organoid size. Augmentation of *SURF1* expression, bezafibrate treatment, or PGC1A induction increases mitochondrial biogenesis, reducing the pathological phenotypes and revealing a potential treatment for LS.^28^ Mitochondrial encephalomyopathy, lactic acidosis, and stroke-like episodes (MELAS) is another mitochondrial disease that causes motor deficits. Winanto et al. developed spinal-cord organoids from patient-derived iPSCs with the causative A3243G mutation to investigate the MELAS neuropathology. Interestingly, motor neurons could not be obtained from mutant iPSCs and were not viable in 2D culture, in contrast to 3D organoid systems. iPSC-derived spinal-cord organoids from MELAS patients show high levels of Notch signaling, resulting in delayed neurodevelopment and neurite outgrowth defects. Inhibition of the Notch-signaling pathway via a gamma-secretase inhibitor DAPT rescued these neural phenotypes, indicating inhibition of the Notch signaling pathway as a potential therapeutic target.^29^

Fragile X syndrome (FXS) is an inherited intellectual neurodevelopmental disorder caused by the loss of *FMRP*, an RNA-binding protein that regulates mRNA translation. Loss of FMRP in iPSC-derived FXS forebrain organoids led to impaired neurogenesis, neuronal maturation, and neuronal excitability. Transcriptomic analysis of iPSC-derived brain organoids from individuals with fragile X syndrome identified altered gene expression related to neurodevelopmental changes. Through enhanced crosslinking, FMRP immunoprecipitation, and high-throughput sequencing or eCLIP-seq, multiple human-specific FMRP mRNA targets, including chromodomain helicase DNA-binding protein 2 (CHD2), and their pathways were identified.^25^ Inhibition of the phosphoinositide 3-kinase (PI3K) pathway with pan-PI3K inhibitors such as LY294002 or selective PI3K inhibitors such as GSK2636771 could reverse the defects in FXS forebrain organoids. Moreover, transcriptome analyses also displayed a large overlap in differentially expressed genes between FXS forebrain organoids and fetal brain tissues, in contrast to the FXS mouse brain, possibly as a result of m^6^RNA modifications, which are present more extensively in human tissues. Hence, human brain organoids serve as better mimics of the FXS-related phenotype, indicating the potential of organoids to reveal human-specific targets for therapeutic intervention.

##### Viral infections

Brain organoids have emerged as useful models for studying viral infections. During the Zika outbreak, models of brain organoids were instrumental in identifying drugs to prevent and combat infection. Screening hPSC-derived cortical neural progenitor cells (hNPCs) with the Prestwick library, containing 1,120 FDA-approved drugs and drug candidates, revealed two candidates, amodiaquine dihydrochloride dihydrate and hippeastrine hydrobromide (HH),^78^ that could inhibit Zika infection. HH was further shown to attenuate microencephaly-related defects in fetal-like forebrain organoids with little toxicity.^44^ In response to the psychological and neurological symptoms associated with SARS-CoV-2, an iPSC-derived brain-organoid model containing microglia was used for modeling infection *in vitro*. Infected organoids showed gene profiles characteristic of neurodegenerative disorders; characteristics included the upregulation of inflammatory genes and synapse engulfment, suggesting a mechanism that could be targeted specifically in microglia to overcome SARS-CoV-2-associated symptoms.^79^ Moreover, hiPSC-derived brain organoids have shown that, displaying selective neurotropism, SARS-CoV-2 preferentially infects specific regions of the brain, including choroid plexus organoids, which display a high expression ACE2 and TMPRSS2 in apolipoprotein-producing cells,^80^ resulting in a breakdown of the blood-brain barrier.^81^ SARS-CoV-2 also displays APOE-isoform-dependent neurotropism, where neurons and astrocytes bearing the *APOE4* mutation as well as co-cultures of neurons with astrocytes are more susceptible to infection in hiPSC brain organoids.^82^ Such organoids provide an alternative to animal models, which require transgenic-mediated expression of viral entry factors such as ACE2 for SARS-CoV-2 infection, although differences in infectivity levels of neurons between different groups warrants more standardization in organoid generation.

Human brain organoids prove useful for studying species-specific viruses, where animal models cannot be used. For instance, Sun et al. recapitulated human cytomegalovirus (CMV)-induced microcephaly by using iPSC-derived brain organoids, which displayed impaired growth, abnormal cortical-layer generation, and dysregulated calcium signaling and neural-network activity. These neurodevelopmental defects could be reversed with the use of neutralizing antibodies 1B2 and 62-11, which target the HCMV envelope pentamer complex,^83^ highlighting the applicability of neutralizing antibodies for HCMV therapy.

##### Fetal-exposure models

In addition to drug screens, organoids can also be used for evaluating the impact of fetal chemical exposure. In a large study, the Swedish Environmental Longitudinal, Mother and Child, Asthma and Allergy (SELMA) study, pregnancy cohort epidemiological data were integrated with data from studies using 2D cell culture, 3D brain organoids, and *in vivo* animal models. To model prenatal exposure, organoids were exposed to groups of endocrine-disrupting chemicals (EDCs) that disrupt hormone action. These chemicals included phthalates, alkyl phenols, and perfluoroalkyl substances. Transcriptional data of human brain organoids exposed to EDC mixtures showed interference with hormonal pathways and dysregulation of gene expression and biological pathways that are causally linked to ASD; such pathways include thyroid, estrogen, and PPAR pathways.^45^ Notably, the cortical organoids used in this study were more sensitive to EDC exposure than fetal progenitors; they showed more differentially expressed genes and more overlap with genes involved in neurodevelopmental disorders. Thus, organoids could become the new gold standard for toxicology, in contrast to fetal progenitors, which are derived from primary human tissue.

In addition to chemical compounds, legal and illegal substances such as alcohol and cannabis impair fetal development. iPSC-derived cerebral organoids modeled fetal-alcohol-spectrum disorders caused by maternal alcohol use during pregnancy. Alcohol exposure in the organoids differentially affected neural cell types; neurons were more vulnerable to apoptosis than astrocytes^84^ and displayed mitochondrial dysfunction and metabolic stress. In a similar study, iPSC-derived forebrain organoids modeled exposure to opiates, cannabinoids, alcohol, nicotine, stress, and maternal immune activation. The study examined the effects of these substances on the proteome, reactome, and metabolome. Exposure to an artificial cannabinoid (WIN 55,212-2) elicited organoid neurotoxicity, inducing apoptosis and DNA damage.^46^

#### Retinal organoids

Retinal organoids are a valuable tool for studying retinal diseases because they can reproduce much of the development and structure of retinal tissue.^85^ Retinitis pigmentosa (RP) is a genetic disorder causing irreversible “night blindness.” However, animal models do not fully recapitulate severe forms of the disease, such as X-linked RP (XLRP), caused by mutations in *RP2.* Animal models for this disease display mild phenotypes, whereas humans exhibit macular atrophy in childhood in some extreme cases. Retinal cells in 2D cell culture that were previously generated do not possess all structural components, such as the inner and outer segments, making them inadequate systems for the study of RP. Retinal organoids (ROs) from *RP2* patient-derived iPSCs were used for modeling a severe form of XLRP,^33^ in which ROs exhibited alterations in Golgi cohesion, Gbeta trafficking, and ciliary trafficking of KIF7. Readthrough drugs such as G418 and Ataluren could reverse the deficits observed in *RP2*-mutant ROs. Mutated *RPGR* iPSC-derived retinal organoids have been applied to model RP.^32^ Both *RP2* and *RPGR* mutant organoid models showed increased photoreceptor cell death, a phenotype not recapitulated in animal models of RP. In addition, the pathologic phenotype in both models was rescued via gene therapy with AAV^33^ or CRISPR-Cas9 reversal of the mutations.^32^ These models illustrate the ability to use retinal organoids to model human-specific RP phenotypes and identify potential therapeutics.

Retinal organoids have also been used to clarify mechanisms behind macular degeneration. The combined application of tumor necrosis factor and heparin-binding EGF-like growth factor induced photoreceptor degeneration via cellular extrusion. Additionally, glial pathologies, dyslamination, and scar formation were also observed. Pharmacological inhibition of PIEZO1, MAPK, and actomyosin prevented degenerative pathology, whereas the activation of *PIEZO1* increased cellular extrusion and degeneration,^47^ presenting viable molecular targets for the therapy of macular degeneration.

### Limitations of current organoid models

Although organoids have significantly advanced disease modeling and drug discovery in the last decade, there is still much room for improvement, and there is a compelling need to bridge the gap between organoids and their endogenous counterparts. Firstly, the gene networks and associations present within organs are not perfectly emulated by organoid systems, and organoid systems often display immature or “fetal-like” behavior. Moreover, some organoid systems exhibit an uncharacteristic stress response, indicating metabolic dysfunction and endoplasmic reticulum (ER) stress, often resulting from inadequate culturing conditions.^86^ Organoids also lack critical components required for maintaining physiologically relevant microenvironment interactions; for example, such components include vasculature and immune cells. Without a suitable microenvironment, organoids are limited by poor nutrient availability, inadequate metabolite removal, and the absence of an adequate immune response, leading to hypoxia and necrosis. Furthermore, organoid research is hindered by a lack of standardization because diverse protocols are used for establishing a variety of organoids, which can lead to sample heterogeneity. Because it is challenging to create identical organoids, designing the appropriate assays for organoid-based drug screening requires critical thinking.

**Figure 2:**
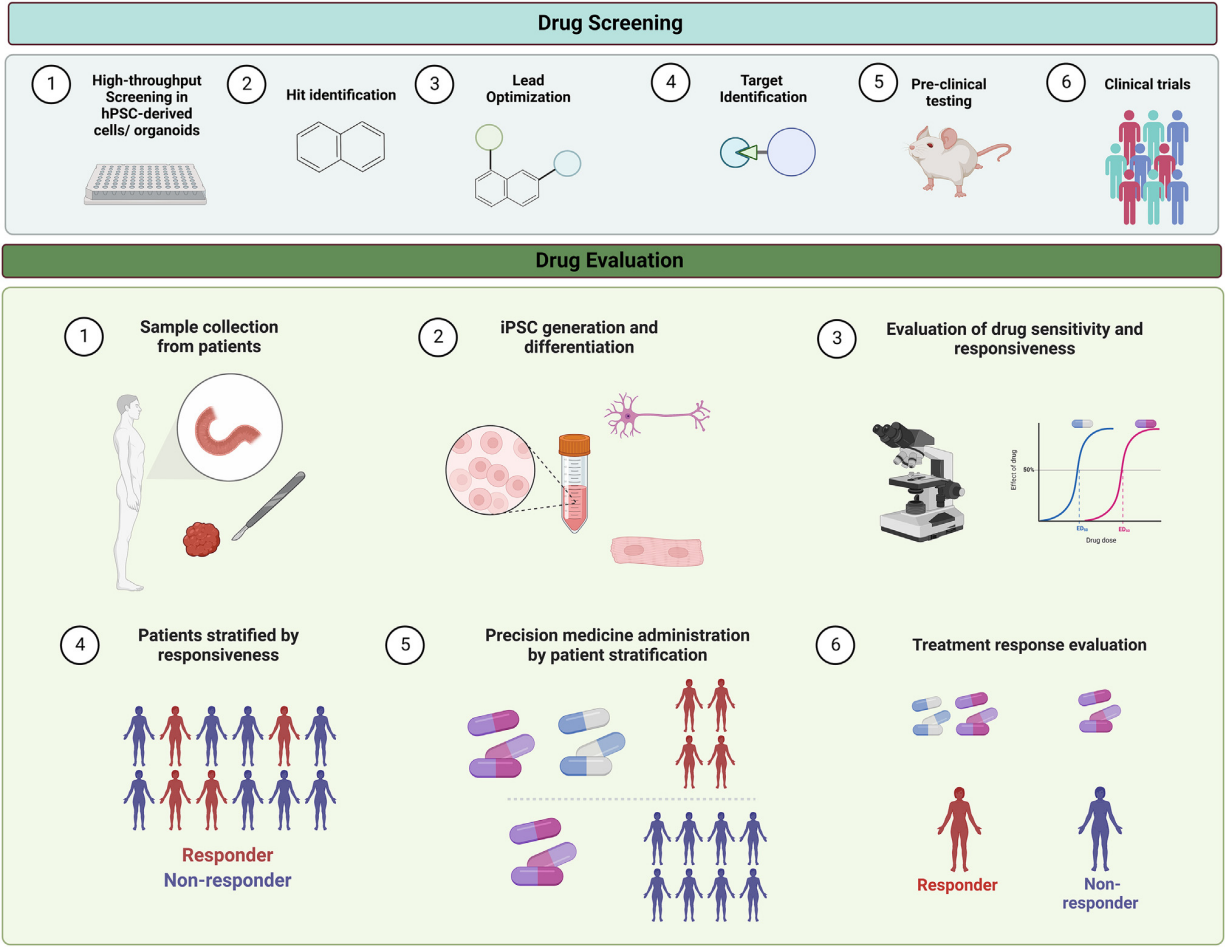
Applications of hPSC-derived organoids for drug screening and evaluation

Ideally, the best model systems should replicate the intrinsic spatiotemporal patterning processes that occur in human tissues and organ; they should display the characteristic markers and phenotypes that are representative of primary tissues and attain adequate maturity. They should be all-encompassing systems and retain key cell-to-cell interactions, an appropriate microenvironment, and suitable mechanical properties, including dynamic flow within the system. These model systems should mimic observed pathologies in humans. In addition, they should be scalable in nature so that researchers can perform high-throughput screening studies to identify drugs that lead to disease attenuation (Figure 2). Organoids, which display most of these key features, can be considered good biological *in vitro* model systems.

Although organoid research has been hampered by a number of limitations, recent developments might help to restore the promise of stem-cell-derived organoids as suitable platforms for drug discovery and evaluation. Much research has focused on improving organoid culturing techniques, including those using different extracellular matrices. For instance, extracellular matrices (ECM) derived from decellularized tissues are able to retain biochemical signatures characteristic of tissue-specific ECM and provide suitable biochemical cues that are able to direct cell fate.^87^ The lack of maturity of organoids makes them especially unsuited for the study of late-onset diseases that present complex phenotypes. Studies have attempted to induce aging in a dish. For instance, one study induced the expression of progerin within iPSCs to mimic aging and recapitulate late-onset diseases.^88^ In another study, the extended culture of organoids led to the recapitulation of late-onset RP.^89^ Thus, exposure to environmental stress, genetic perturbations of aging-associated factors, and extended culture accelerate the development of aging-associated phenotypes in hPSC-derived organoids. Other studies have targeted the inclusion of microenvironment interactions within organoid systems. Co-culturing with endothelial cells, co-differentiation with mesodermal progenitor cells, and mechanical stimulation have all been investigated as means of incorporating vasculature within organoids.^90^ In a recent study, the transplantation of HIOs under the kidney capsule of mice with a humanized immune system resulted in the generation of HIOs containing immune cells resembling lymphoid follicles.^91^ Such systems are especially beneficial for the investigation of the immune crosstalk in a range of diseases, including both infectious diseases and non-infectious diseases such as cancer. As a result, studies utilizing such organoids can provide critical insights on drug development and predictive drug response in patients.

### Two-dimensional cultures versus organoid models

Over the last several decades, 2D cultures have been the preferred model for drug screening because of their low maintenance cost, easy handling, and high expandability, which enable high-throughput, high-contentstudies. However, the existing limitations of 2D systems have become increasingly apparent, necessitating a shift toward more physiologically relevant 3D organoid systems.

One of the primary limitations of 2D systems is their inability to adequately mimic the physiological environment, particularly the lack of a microenvironment and multicellular interactions present in organoid models. As a result, 2D models might not accurately emulate complex disease phenotypes, which can limit their predictive power in drug screening assays. Furthermore, the transcriptional signatures of 2D models often diverge widely from those of their endogenous counterparts and 3D organoid systems, further highlighting their limitations.

However, the use of organoids for drug screening also has challenges. Organoids can be more difficult to culture and maintain than 2D cultures, and the cost of generating and screening organoids can be high. Additionally, the heterogeneity of organoids can complicate data interpretation. Despite the challenges associated with organoid-based drug screening, the shift toward these more physiologically relevant models is likely to continue. Ultimately, the choice of model will depend on the specific research question, resources available, and desired level of physiological relevance.

### Advances in organoid-based technology

**Figure 3:**
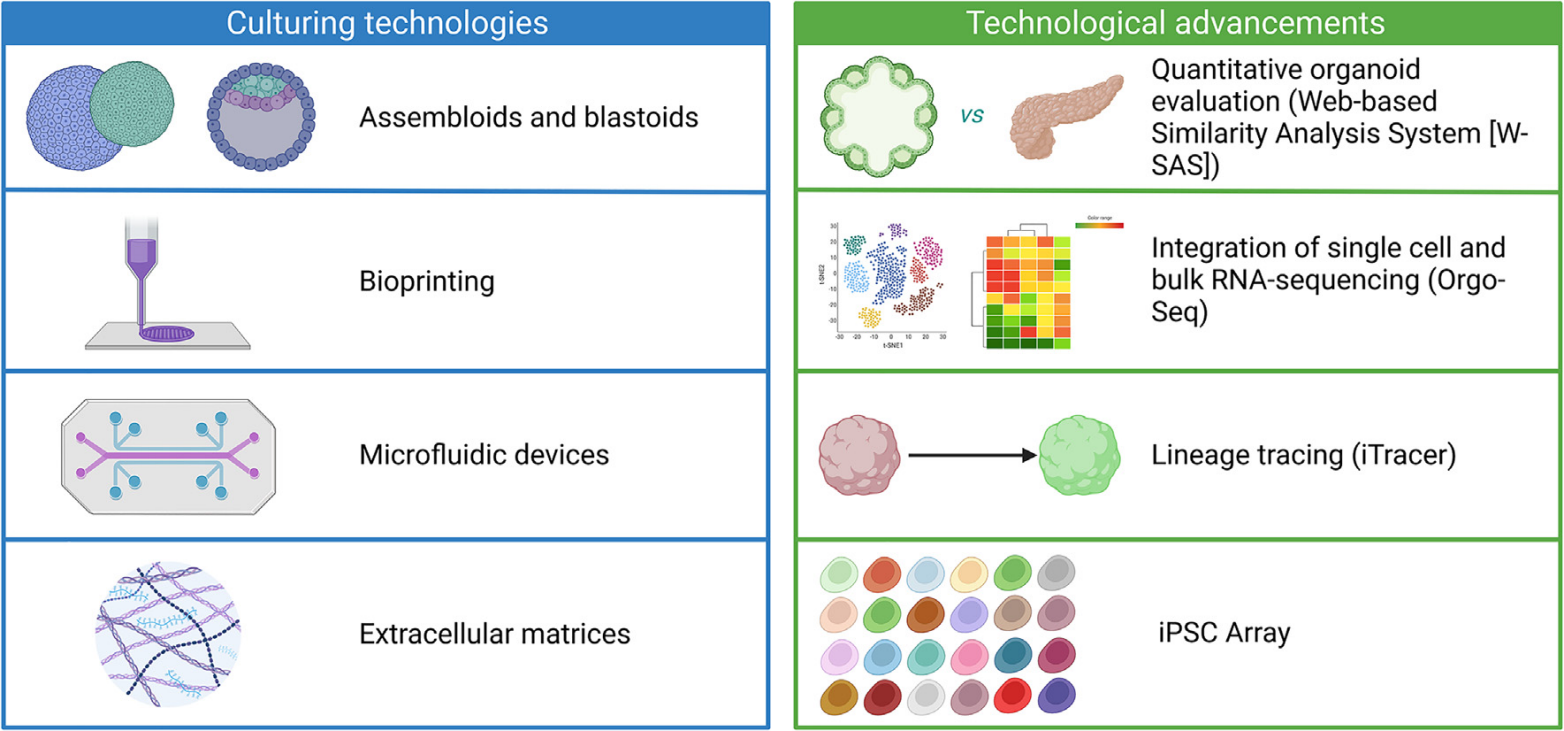
Innovations in hPSC culturing technologies and technological advancements

Current organoids have not yet become perfect model systems; their several limitations include heterogeneity, immaturity, the absence of a suitable microenvironment, and the lack of suitable mechanical properties. Several transformative developments in organoid research have been geared toward eliminating the drawbacks of using organoid model systems (Figure 3).

Assembloids, which are spatially organized combinations of different cell types, have proven to be useful in gaining a better understanding of processes where spatial organization is crucial.^92^ For instance, an assembloid of pericyte-like cells (PLCs) integrated into cortical organoids supports astrocyte maturation and allows for SARS-CoV-2 infection and replication, providing a valuable experimental model for studying neural infections.^82^ In addition, forebrain assembloids composed of cortical and ventral forebrain components have uncovered cortical interneuron migration defects in the neurodevelopmental disorder Timothy Syndrome and have further elucidated the molecular basis for this defect.^93^^,^^94^ Recently, there has been a significant effort to develop blastocyst-like organoids called “blastoids,” which recapitulate blastocyst development, including the sequence and timing of the formation of trophectoderm, epiblast, and primitive endoderm. Variety protocols have been developed to optimize the transcriptional, spatial, and developmental similarity of blastoids to human blastocysts,^95^^,^^96^^,^^97^ making them a powerful model for studying embryonic implantation and early human development.^98^ With cellular components from different germ layers arranged in their appropriate spatial location, these complex organoids have the ability to better mimic human physiology and disease states. Thus, they offer a more accurate representation of drug response and toxicity. Moreover, the ability to incorporate multiple cell types and mimic spatial organization offers a powerful platform for modeling complex diseases and testing therapeutic interventions. With ongoing improvements in organoid technology, including the incorporation of vascularization and immune cells, as well as the development of more standardized protocols, the use of blastoids and assembloids in drug screening could become more common in the future.

Additional technologies improving organoid generation and physiological relevance include 3D bioprinting of iPSC-derived cells, which allows for the high-throughput generation of kidney^99^ and neural organoids^100^ with reduced variability and thus improves their suitability for large-scale screening. Microfluidic devices that mimic cerebrospinal and interstitial fluid flow in organoids improve survival and reduce variability. Indeed, organ-on-a-chip platforms are emerging technologies that are highly beneficial for drug screening as a miniaturized system that enables control over physical parameters such as fluid flow. Moreover, multiple organoids can be incorporated into a single chip, which presents a more representative model system.

Computational tools have played a significant role in enhancing the data obtained from organoids. However, variation among organoids limits their utility in biomedical applications such as drug screening. To better calculate and understand the similarity of human organoids to their intended human organ(s) *in vivo*, Mi-Ok Lee et al. developed a quantitative calculation system utilizing the GTEx database. This system uses user-provided RNA-sequencing data from organoids to predict organoid similarity to target organs via a web-based user interface called Web-Based Similarity Analysis System (W-SAS).^101^ Elaine T. Lim et al. developed Orgo-Seq, a framework to integrate bulk and single-cell RNA-sequencing data from already sampled cerebral organoids and postmortem brain tissue, allowing for the identification of cell-type-specific driver genes associated with neurodevelopmental disorders.^78^ Organoids also provide a valuable tool for studying human development. He et al. established iTracer, a lineage recorder that combines barcodes with inducible CRISPR-Cas9 scarring to trace individual clones in organoids during development. Additionally, iTracer can be expanded to link clones with location information in organoids or combined with genetic perturbations for simultaneous lineage tracing and genetic knockouts in mosaic organoids.^102^ Recently, an iPSC-based genome-wide association study identified the genetic variants, regulatory regions, and genes associated with ZIKV infections.^103^ In addition, hPSC collections can also be used to model gene-environment interactions in post-traumatic stress disorder^104^ and upon environmental toxin exposure.^105^ Overall, the combination of computational biology and organoid biology will continually provide novel tools to dissect biological questions.

### Concluding remarks

Advancements in hPSC technologies over the last two decades have shifted toward 3D culturing methods, such as organoids, blastoids, assembloids, and organs-on-a-chip. Today, hPSC-derived organoids are a powerful tool for chemical screening and facilitate pre-clinical and clinical discovery. The organoid provides a model complementary to traditional systems, such as animal models, primary cells, and cancerous cell lines. Recent advancements in gene-editing techniques, such as CRISPR-Cas9 and prime and base-editing, have provided new means to study genetically complex diseases. As technological advancements continue to bridge the gap between organoids and primary tissues, organoids and organ systems can now be assembled to more closely simulate human organs and to come complete with finely tuned and integrated cell-signaling networks that enhance their responsiveness to non-genetic factors, such as exposure to toxins, chemicals, and metabolites. Furthermore, due to the expression of key receptors, hPSC-derived organoids are now being applied to study viral infections, including viral tropism, host response, and immune-mediated host response, as well as for drug evaluation. However, current organoid models still have drawbacks, such as developmental immaturity and heterogeneity. Thus, it is critical to choose appropriate assays for drug screening and evaluation. Recent developments in experimental tools and technologies, including organ-on-a-chip technologies, iPSC banks, and new computational and single-cell sequencing tools, are transforming hPSC-based organoid systems into advanced systems that will be routinely used in drug discovery.
